# Advanced Architecture of On-Board Multiplex Information Exchange System to Increase Flight Safety

**DOI:** 10.3390/e24111582

**Published:** 2022-11-01

**Authors:** Viktor Dashkiiev, Yuriy Povstenko

**Affiliations:** Department of Mathematics and Computer Sciences, Faculty of Science and Technology, Jan Dlugosz University in Czestochowa, al. Armii Krajowej 13/15, 42-200 Czestochowa, Poland

**Keywords:** digital information system, multiplex information exchange system, processing unit, information exchange subsytem (IXS), parallel computations, avionics

## Abstract

Issues of aviation flight safety conducted with an on-board information exchange system are discussed. The system information analysis method is used. An analysis of in-flight hazard sources and the means of their elimination from the information exchange point of view are carried out including the influence of the information exchange system’s parameters, work algorithm and architecture. The value of information exchange speed on safety in dynamic flight conditions is discussed. A modification of architecture and the information exchange system algorithm are proposed to increase the exchange speed and the whole system’s effectiveness based on parallel processing as well as to enhance the probability of successful elimination of an emergency situation.

## 1. Introduction

The widespread use of digital information systems increases the importance of the issues of ensuring the reliability and uninterrupted operation of their work. This is especially true for critical information systems, on which the preservation of life and health of people depends. First of all, this concerns aviation airborne systems. Their failure causes severe flight accidents with numerous casualties and great material damage. For example, it is enough to recall the crash of the Russian Sukhoi Superjet airliner at Sheremetyevo Airport on 5 May 2019 or the Boeing-737 MAX near Addis Ababa, Ethiopia, on 10 March 2019. In both cases, the cause was a failure of the aircraft’s electronic control system. The responsibility of such systems is supplemented by specific working conditions in aviation. This includes, firstly, the transience of physical processes which places extremely stringent requirements on the speed of the information system and, secondly, significant and continuously growing volumes of information that are processed and exchanged by on-board electronic systems. All this is superimposed on the standard requirements for aviation to minimize the mass, volume and energy consumption of such systems.

Thus, it is obvious that the subsystem of information exchange is critical from the point of view of reliability and security. Its functions include, as a rule, interrogating the sensors of measuring systems and determining the order of such an interrogation, collecting and processing information, if necessary, converting information packets from one to another exchange protocol and, finally, forwarding information packets from the source subsystems to the user subsystems. It is apparent that the discussed subsystem of information exchange is a nodal, critical point at which all information flows converge. Its failure entails the termination of the functioning of the entire on-board information system as a whole, with corresponding material consequences. This problem was under the scrutiny of developers from the very beginning of the implementation of digital systems in aviation (see, for example, [1,2,3]).

The reliability analysis of a repairable data transmission system was performed in [4], where the methodology was proposed to evaluate the reliability of the entire system in the case of failure of its components. A comprehensive review of fault diagnostics for Industrial Cyber–Physical Systems, including studies from aerospace and avionics, was presented in [5]. Methods of data-driven fault diagnostics were reviewed in [6]. The structure and logical description of a model of reasonable redistribution of diagnostic functions for increasing the reliability of functioning of the information management system were elaborated on in [7].

Let us review the main features of aviation on-board digital information systems. The key features, while they are used in the aerospace industry, are severe mass, volume and layout restrictions as well as limitation on energy consumption. Due to the specifics of the main product in aviation, both the total number of signal cables (weight restrictions) and the number of places where they can be laid (layout constraints) are strictly limited. That is why the so-called multiplex information exchange systems are almost universally used now. The general ideology of such systems is to use a limited number of signal cables (buses) to which all components of the on-board information system, transmitters (measuring sensors) and receivers (various aircraft systems that need information) are connected. At the same time, at each moment, there is a single working signal line.

Electromagnetic compatibility problems may also arise in addition to compatibility at the information level if such a system is implemented in a limited volume on board an aircraft (electrical noise in the signal line, induced currents from electromagnetic noise, etc.). This is just the opposite situation to commercial mobile networks, where communication subscribers are far from each other at a sufficiently large distance and the issue of interference of physical fields is much less important. In addition, there are many requirements of standards and regulations, quite strict and conservative in the case of aircrafts.

The issues of electromagnetic interference caused by the joint operation of on-board navigation and communication equipment were considered in [8], and an algorithm for a refined model of this phenomenon was proposed. Electromagnetic interference caused by the joint operation of avionics and satellite navigation systems was examined in [9], where criteria and methods for calculating the impact of such interference on flight safety were proposed and promising ways to improve safety were suggested. A report on full-scale experiments on electromagnetic compatibility with all main types of on-board radio-electronic equipment was presented in [10], with comprehensive experimental data and a theoretical analysis of the results. A large array of issues related to the influence of the quality of maintenance of on-board radio-electronic equipment as well as the methods of its technical diagnostics and existing regulatory documents on fail-safety and, consequently, on flight safety are covered in [11].

The on-board digital system of the aircraft consists of (see [12]): (a) sensors, measuring devices and systems for evaluating the parameters of the external environment and the movement of the aircraft, determining its location in space as well as internal parameters of the state of the aircraft and its systems; (b) aircraft control systems; (c) information processing and decision-making systems; (d) external signal exchange systems (communication, radio navigation, satellite navigation); (e) communication lines; (f) on-board power supply systems.

In this case, the key issue is the organization of information exchange in which the high speed of signal exchange and high information performance of the entire system are combined with high reliability. This is achieved by introducing a separate information exchange subsystem—the information exchange subsytsem (IXS). The IXS is connected to the signal lines (data buses) along with all other subscribers, but differs from them as it controls the rest of the information process, issuing a command to all other components to transmit and receive signals.

Let us analyze the existing publications on the impact of various aspects of reliability and characteristics of the on-board information exchange system on flight safety. Here, first of all, it is necessary to distinguish between the properties of the IXS, which are common with all other avionics (in physical, structural and other senses) and the specific functional properties of the IXS as a unique on-board subsystem that has no other analogues. The IXS is an ordinary on-board computer complex from the point of view of the general properties of avionics, which includes all the components characteristic for such systems: power, computing devices, signal lines and software. A huge backlog has been accumulated, both scientific works and regulatory documents and standards according to the general properties of avionics. In [12], the behavior of the on-board information system in the event of failure of any of the components of the information exchange subsystem, either processing unit or signal line, was studied. The architecture of the information exchange subsystem was analyzed, which is the most effective in terms of safety in the case of failure; reconfiguration of the subsystem in the case of failure of the processing unit or signal line was investigated as well as algorithms for self-control and mutual control of the components of this subsystem.

The operation of the aircraft navigation system under conditions of signal reception errors was studied in [13] concerning the information aspect; algorithms for modeling physical and information processes under these conditions were given and circuit and algorithmic solutions aimed at improving safety were proposed. Emphasizing software quality, the influence of errors made during software development on the probability of a prerequisite for a flight accident was considered in [14]. The main emphasis was placed on the errors made in the logic of computer programs and measures were proposed to reduce their impact. An analysis of the impact on flight safety of various categories of errors in software was carried out in [15], and methods for testing software were proposed for the detection of complex errors and dynamic errors, the most difficult for their detection and correction.

Similar structural and algorithmic solutions for digital information systems were considered in different fields (mechatronics, oil industry, etc.) [16,17,18]. Though the paper [17] concerns the subsea oil industry, the authors propose a method for analyzing the reliability of the information exchange systems using stochastic Petri nets which may be useful in other applications. In [18], the model of mechatronic system operation and reliability was developed to predict the critical situations.

Based on the analysis of the probability of failure-free operation of redundant information systems [19], we can conclude that in practice any information exchange system, regardless of its architecture, should be considered as a system with a loaded reserve. The algorithm should be applied specifically for a system with a loaded reserve when calculating the characteristics of its trouble-free operation.

The objective of this paper is to find circuit and algorithmic solutions that will improve the safety level of aircraft operation by refining the system of on-board information exchange. An analysis of in-flight hazard sources and the means of their elimination from the information exchange point of view are carried out, including the influence of information exchange system parameters, work algorithm and architecture. The value of information exchange speed on safety in dynamic flight conditions is discussed. The modification of architecture and algorithm of information exchange system is proposed to increase the exchange speed and the whole system effectiveness based on parallel processing as well as to enhance the probability of elimination of an emergency situation.

The rest of the paper is organized as follows. In Section 2, we present the time layout of the operating cycle of the on-board information exchange system. In Section 3, we describe the proposed improvement of the on-board information exchange system which allows us to increase the probability of successful elimination of emergency situation in an aircraft and thus to increase flight safety. Additional discussion is presented in Section 4. Finally, we conclude this paper in Section 5.

## 2. Time Layout of the Operating Cycle of the On-Board Information Exchange System

Now we will analyze the time layout of the process of occurrence and development of an emergency situation on board an aircraft and the reaction of the digital control system on it. Consider the full operating cycle of the information exchange system. The cycle begins with a service signal exchange block, the duration of which is $t_{\mathrm{sseb}}$, and during which the signal transformation units carry out an internal exchange of signals: self-monitoring and mutual control, synchronization, signaling the failure of on-board systems or signal lines, etc. After the completion of the service signals, the exchange block begins the polling cycle of signal sources. At each step, for example, for the *i*th transmitter, a service signal exchange pack is first transmitted/received, the duration of which is $t_{\mathrm{tssep}}^{i}$, and which includes checking the transmitter’s serviceability, a command to start transmission, if necessary, sending corrective settings, etc. Further, the *i*th transmitter sends a signal packet of duration $t_{t}^{i}$, after which the (i+1)th transmitter is polled, which also includes a service signal exchange pack of duration $t_{\mathrm{tssep}}^{\left(i+1\right)}$ and reception of a signal packet of duration $t_{t}^{\left(i+1\right)}$. This is repeated for *N* receivers in the system. After the completion of the polling cycle for transmitters, the working information exchange unit processes the received signal packets with a duration of $t_{\mathrm{proc}}$. During this, for example, the conversion of signals from one exchange protocol to another is performed, in cases when they differ; as well as repacking of signals, in cases when the format of signal packets issued by transmitters differs from what is required by receivers. For example, different consumers require different parts of the common package of signals. At this time, the reception or transmission of signals is not performed by any subscriber, since the working information exchange unit is busy and cannot manage the exchange of information. The signal packet transmission cycle to consumers begins when processing time $t_{\mathrm{proc}}$ has elapsed. Similarly, the transmission of a packet to the *j*th receiver includes the transmission/reception of a service signal exchange pack, the duration of which is $t_{\mathrm{rssep}}^{j}$, and the transmission of a signal packet with a duration of $t_{r}^{\left(j+1\right)}$. This is repeated for the *M* receivers in the system. The total duration of one full cycle of the information exchange unit will be:

(1) $$T=\sum_{i=1}^{N}\left(t_{\mathrm{tssep}}^{i}+t_{t}^{i}\right)+\sum_{j=1}^{M}\left(t_{\mathrm{rssep}}^{j}+t_{r}^{j}\right).$$

Next, we will consider the process of occurrence and development of an emergency situation on board an aircraft and the reaction of the control system to it, with a layout in time. Suppose that an emergency situation has begun at time $t_{0}$, the identification sign of which is the value of some parameter $P_{k}$ going beyond the allowable limits. This parameter is measured by the *i*th sensor. We assume for simplicity that the sensor has no inertia and its own response time is zero. That is, the fixation of a critical change in the parameter $P_{k}$ occurred at the time of its actual occurrence $t_{0}$. The most unfavorable variant assumes that the moment $t_{0}$ came immediately after the *i*th sensor was investigated and transmitted the previous, still normal value of the parameter $P_{k}$. The new, critical value of the parameter $P_{k}$ can be transmitted to information consumers only after the information exchange system performs one complete cycle and the signal line is again released for the *i*th sensor. Therefore, the critical value of the parameter $P_{k}$ will be transmitted at time $t_{0}+T$. If we assume the most unfavorable case, when the transmitter is the first in the transmission queue and its receiver is the last in the receiver queue, then the moment of delivery of the critical value $P_{k}$ to the receiver will be circa $t_{0}+2T$. Further, we assume that an intelligent aircraft control system, either a human pilot or artificial intelligence, needs a period of time $t_{\mathrm{urt}}$ (understanding reaction time) to recognize a critical situation and develop an appropriate command to eliminate it. Given that the intelligent control system is a participant in the information exchange, the control command, by analogy with the above, will be delivered to the end user, taking $t_{\mathrm{urt}}+2T$ after an alarm enters the intelligent control system. Since the control of an aircraft is associated with mechanical actions, each aircraft control is characterized by a delay in the execution of a command, due to both its own inertia of the actuator and the natural resistance of the air or internal environments of the aircraft. Therefore, the execution of each control command, whether it is opening/closing the fuel supply valves, turning the rudders, retracting/extending flaps and so on will take a period of time $t_{\mathrm{exec}}$ upon the moment when the control command is received. This value varies depending on the purpose and design of the control as well as on the conditions of its operation. However, any aircraft control cannot directly change the state of the aircraft and/or the environment, but it creates only some mechanical or other functional impact. The aircraft and its systems require a reaction time $t_{\mathrm{art}}$ (aircraft reaction time) to work out the control input.

As a result, the full control cycle $T_{\mathrm{contr}}$ of the aircraft, from the appearance of an external disturbance to the execution of a command to parry it, will be:

(2) $$T_{\mathrm{contr}}=2T+t_{\mathrm{urt}}+2T+t_{\mathrm{exec}}+t_{\mathrm{art}}=t_{\mathrm{urt}}+t_{\mathrm{exec}}+t_{\mathrm{art}}+4T.$$

In any case, a one-time control action of the control system is almost never performed in one $T_{\mathrm{contr}}$ cycle in reality. The fact is that it is practically impossible to calculate the magnitude of the control action with sufficient accuracy under conditions of uncertainty in the state of the air environment and large errors in determining the kinematic and dynamic parameters of the movement of the aircraft itself. Control-with-correction is implemented in almost all cases. At the same time, a command is consciously given to the executive body of the control system to perform a control action that is somewhat larger or somewhat smaller than what is actually required. A “clarifying” corrective action will be required after its implementation, much smaller in amplitude than the previous one. The duration of the information exchange subsystem cycle for the case of an unstable aircraft becomes one of the decisive factors determining flight safety.

Let us analyze the terms of the equation

(3) $$T_{\mathrm{contr}}=t_{\mathrm{art}}+t_{\mathrm{exec}}+t_{\mathrm{urt}}+4T.$$

It is known from practice that the response time $t_{\mathrm{reac}}$ of an aircraft to a control action depends on its mass, inertial characteristics and aerodynamic layout and, therefore, has practically no reserves for improvement. The response time of the controls for the execution of the $t_{\mathrm{exec}}$ command lies outside the field of computer science and therefore is not discussed in this paper. The command generation time $t_{\mathrm{com}}$ also does not contain visible reserves for its reduction. It can be assumed in the case of the use of an artificial intelligent system that its performance is determined by the current level of microelectronics technology, so a further improvement can only occur aside the significant progress in this industry. In the case of aircraft control by a human pilot, the speed of the latter is limited by biological restrictions, and also does not contain any growth reserves. The only way to reduce the cycle time of the control system is to reduce the cycle time of the information exchange subsystem. Taking into account the peculiarities of its functioning, reducing the duration of its cycle *T* can be achieved by changing the architecture and algorithm of the system.

## 3. Proposed Improvement of Aviation On-Board Digital Information System

**Figure 1:**
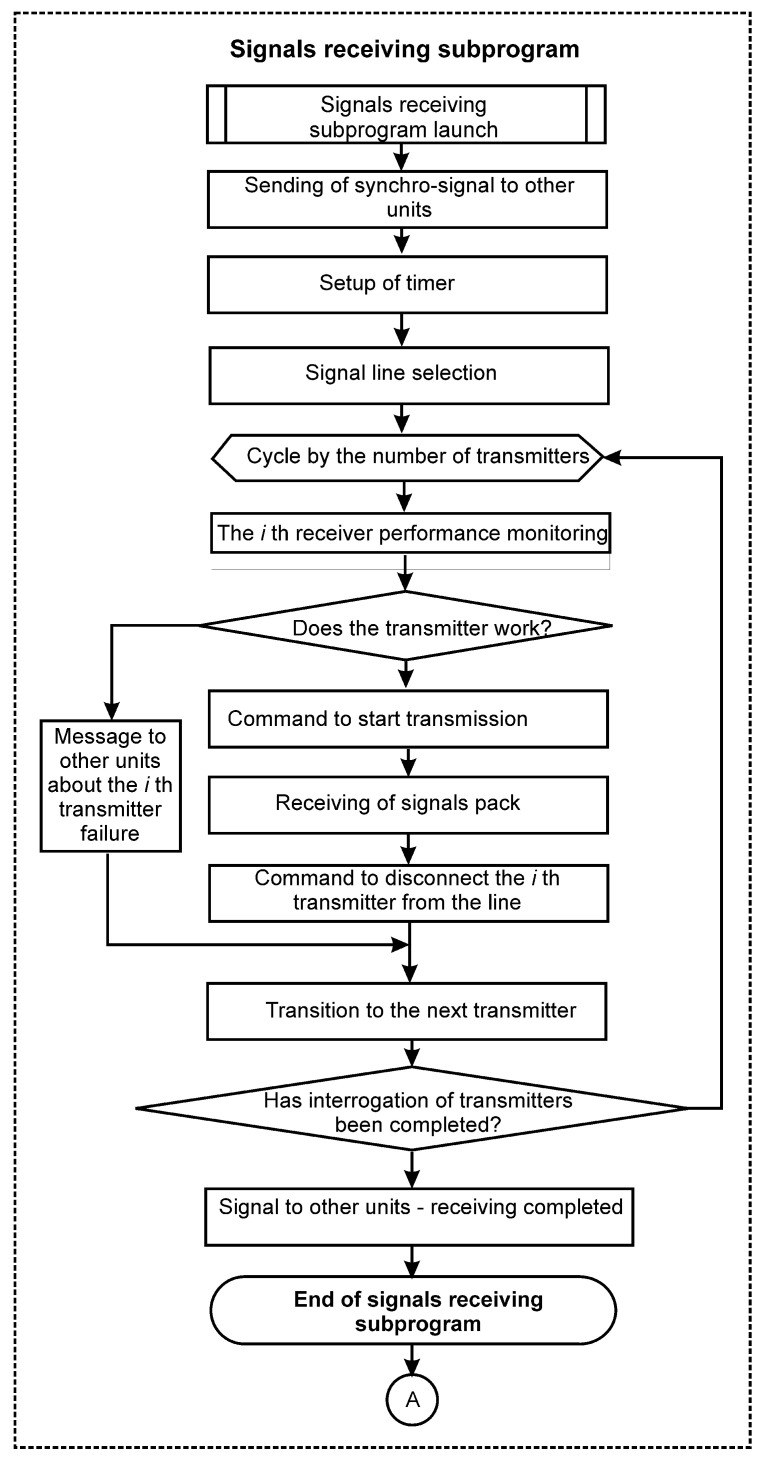
The work block-scheme for one of the units in the proposed airborne information system. **Start**—subprogram of signal receiving.

**Figure 2:**
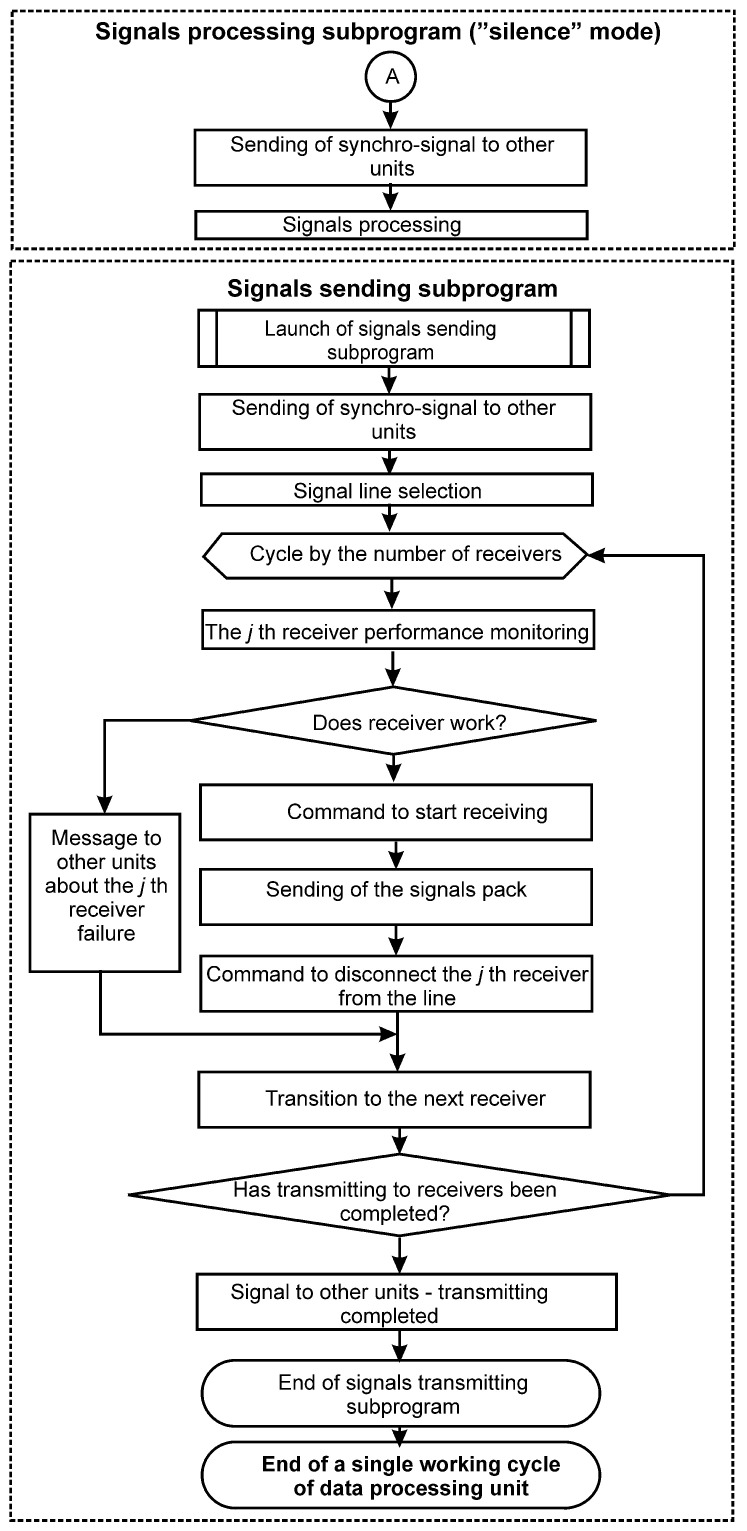
The work block-scheme for one of the units in the proposed information exchange system. **End**—subprograms of signal processing and signal transmitting.

Aviation on-board digital information systems can be divided into classes of signal consumers (receivers), signal sources (transmitters) and signal transmission lines. The information exchange system algorithm proposed in the present paper is shown in the form of block diagrams in Figure 1 and Figure 2. Each of *N* IXS units is allocated to one of the signal buses for exclusive use during each complete IXS cycle and the (N+1)th bus remains redundant, i.e., unused. Moreover, the allocation of tires is cyclically shifted at each subsequent point in time. That is, if at the time moment $t_{i}$ an IXS unit $B_{i}$ was allocated to the line $L_{j}$ and the line $L_{k}$ remained in reserve, then at time $t_{i+1}$ ($t_{i+1}=t_{i}+T_{t}$), the line $L_{j+1}$ will be allocated to the unit $B_{i}$ and the line $L_{k}$ will be already reserved by the line $L_{k+1}$. This eliminates the possibility that the line, permanently allocated as a backup, will be damaged and this damage will remain undetected until the moment when it is needed.

The system operates according to the following algorithm. At time $t_{i}$, the IXS unit $B_{i}$ sends a ring synchronization signal to all other units $B_{i+1},\;B_{i+2}$, etc., to reset the watchdogs and then the number of the signal line $L_{j}$, which it occupies. After that, it starts cyclically interrogating the transmitters $T_{i}$ ($i=1,\ldots ,K_{T}$, where $K_{T}$ is the total number of transmitters). At the same time, it alternately exposes the address of the next interrogated device $T_{k}$ to the $L_{j}$ allocated to it, then performs a health check $T_{k}$ and sends a transmission enable bit. After that, the transmitter $T_{k}$ issues a sequence of signals to the line that make up its message. After reading the message, $B_{i}$ puts the address of the next polled device $T_{k+1}$ on the bus. The data reading cycle is repeated.

At time $t_{i+1}=t_{i}+T_{c}$, the previous cycle of unit $B_{i+1}$ is completed, conditionally “lagging behind” the unit $B_{i}$ by the amount of the phase shift. The unit $B_{i+1}$ sends a synchronization signal around the ring to all other units $B_{i},\;B_{i+2}$, etc., as well as the number of the signal bus $L_{j+1}$, which it occupies. After that, it starts polling the transmitters $T_{i}$ ($i=1,\ldots ,K_{T}$), only on the line $L_{j+1}$ allocated to it. In the meantime, the unit $B_{i}$ completes the polling of transmitters, then processes the information received from them such as performing mathematical operations embedded in the program, converting from one exchange protocol to another, etc. Then, according to the algorithm described above and via the same data bus $L_{j}$, information packets are sent to the corresponding receivers $R_{l}$ ($l=1,\ldots ,M_{R}$, where $M_{R}$ is the total number of receivers).

At time $t_{i+2}=t_{i}+2T_{c}$, the previous cycle of the unit $B_{i+2}$ is completed, conditionally "lagging behind" the unit $B_{i}$ by two phase shifts. The unit $B_{i+2}$ sends a synchronization signal around the ring to all other units $B_{i},\;B_{i+1}$, etc., as well as the number of the signal bus $L_{j+2}$, which it occupies. After that, it starts polling transmitters $T_{i}$ ($i=1,\ldots ,K_{T}$), only on the $L_{j+2}$ line allocated to it. Then the process is repeated according to the above algorithm. The line $L_{k}$ remains idle (backup). After the completion of the full cycle of the entire IXS, at time $t_{k}=t_{i}+N\cdot T_{c}$, a phase shift is performed on all signal lines. The unit $B_{i}$ is allocated to the bus $L_{j+1}$, the unit $B_{i+1}$ is allocated to the bus $L_{j+2}$, and so on. The line $L_{k+1}$ is already assigned as a backup.

Suppose that the on-board information exchange subsystem (IXS) consists of *N* units (let N=3 as an example). Signal buses are laid, to which sources and consumers of signals are connected for receiving and transmitting signals as well as the IXS. The number of signal lines is assumed to be N+1 to ensure reliability (let N+1=4 as an example). Moreover, each receiver, each signal transmitter and each IXS unit is connected to all N+1 buses. In addition, *N* IXS units are connected to each other in a ring for the exchange of service information such as self-control, synchronization, etc.

**Figure 3:**
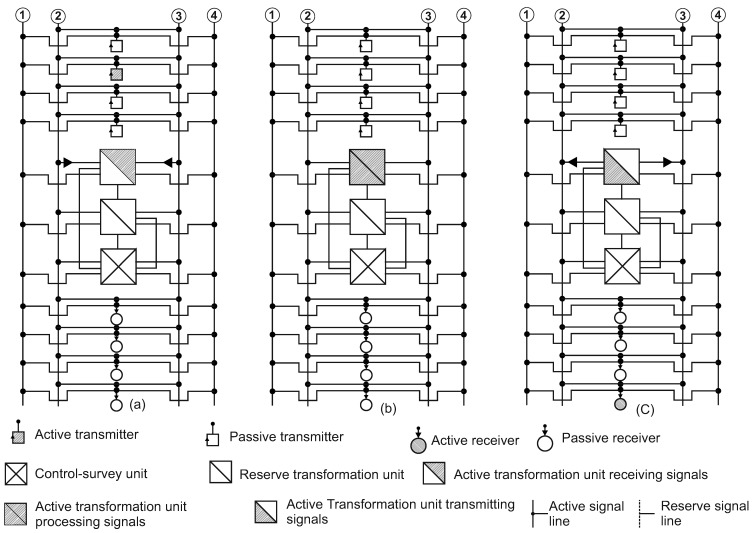
The structural scheme of the existing information exchange system: (**a**) a phase of receiving signals from data sources; (**b**) a phase of processing received signals; (**c**) a phase of transmitting processed data to consumers of information.

**Figure 4:**
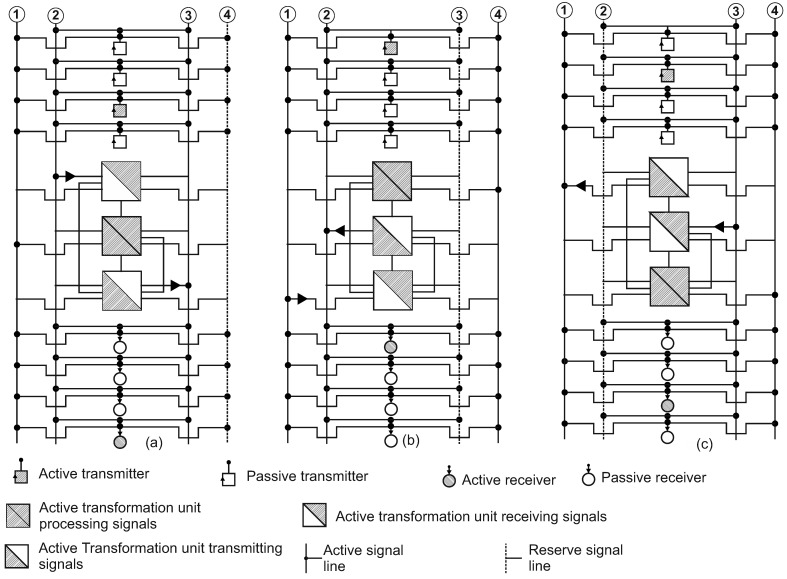
The structural scheme of the proposed information exchange system: (**a**) a phase of receiving signals from data sources; (**b**) a phase of processing received signals; (**c**) a phase of transmitting processed data to consumers of information.

A structure scheme of the existing airborne information exchange system is shown in Figure 3. Similarly, Figure 4 shows a structure scheme of the advanced system proposed in this paper. It should be emphasized that the original architecture is implemented on the vast majority of commercial and military aircrafts; the changes can only concern the reduction of certain components. The reliability of components such as sensors, actuators and signal transmission lines is ensured by duplication of the components themselves or their functions. Some of them, for example, sensors and communication lines, operate in parallel and independently of each other. Alternative ones are actuating devices and operate according to the scheme “one is working, doubles are idling”, and they are switched by a signal from an external control system, for example, if the first instance fails. Abstracting from the design and functional features, we note that in this article the structure of systems can be conditionally called hierarchical [19]. The hierarchical structure includes several (two or more) autonomous computing devices that are the same or differ in design and characteristics. In this case, the working function of the information exchange subsystem is performed by the first (conditionally the “head” one, according to the information flow) Processing Unit (PU). The second and subsequent PUs control the previous device(s). The last ones take over the working function of the information exchange subsystem in a case of failure of the previous one. The operation algorithm of such a structure is sequential.

Such a structural scheme is implemented in practice and can be called hierarchically interleaved. In a generalized form, it is presented in Figure 3 (a variant consisting of three identical PUs is shown). It can be seen from Figure 3 that each source and each receiver (consumer) of information are connected in parallel to four communication channels, two (main and backup) for each of the two head or functional PUs. Conditionally, behind them there is the third, control PU (supervisor). The two head PUs change their roles cyclically: in turn, on each subsequent work cycle, first one, then the second PU perform the functions of the head and another performs the functions of a supervisor.

A “reinforced” or parallel-series version of such a circuit is also possible. In this case, both head PUs are functional simultaneously and their work is strictly synchronized. At the same time, they expose information packets for transmission to all four lines. The third PU (supervisor) reads packets from all four lines and compares them with each other. The difference in the contents of the packages is considered as a sign of failure, which implements an additional layer of security.

Initially, the subroutine for polling signal sources is executed (Figure 3a). According to the algorithm, the functional (head) PU sequentially polls subscribers by setting the channel opening bit on the line of the next transmitter and channel closing bits on the lines of other subscribers. Then a packet of information (frame) is received from this subscriber, after which a closing bit is set on its line. This sequence includes additional service operations if necessary, for example, monitoring subscriber health, etc. The sequence is then repeated for the next transmitter in the queue. Note that all other sources and all signal consumers are forced to be inactive at the moment of operation of the next transmitter. Upon the completion of the subroutine for polling transmitters, the second subroutine is executed according to the algorithm—namely, signal processing. The PU handles information: converting according to the required exchange protocol, etc. (Figure 3b). Only the IXS works during this period, while all the receivers and transmitters on the board cannot receive and send signals, i.e., they are forced to be inactive. Upon completion of the signal processing subroutine, a subroutine of newly formed information packets to consumers–receivers is executed (Figure 3c). All other consumers and all signal sources are forced to be inactive at the moment of operation of the next transmitter. All these operations together constitute the working (functional) cycle of the PU.

The unit diagram of the proposed solution is shown in Figure 4. Figure 4a–c are given according to the phases of the conditionally “first” or “head” unit of IXS. Note that in the proposed architecture all the IXS units are equal and are involved in the work at the same time. The *i*th complete cycle of the system is shown. In phase I (Figure 4a), unit #1 cycles through the serial reception of signals from transmitters on the signal line #2. At the same time, unit #2 connected to the signal line #1 performs internal processing of received signals (“silent” mode), and unit #3 connected to signal line #3 performs a serial signal transmission cycle to receivers. Signal line #4 remains free (in reserve). In phase II (Figure 4b), unit #1 processes the received signals, unit #2 transmits the processed signals and unit #3 performs a transmitter poll cycle. In phase III (Figure 4c), unit #1 transmits the processed signals, unit #2 performs a polling cycle for the transmitters and unit #3 processes the received signals.

On the (i+1)th full cycle of the system, the working signal lines are changed (Figure 4b). Unit #1 is connected to signal line #4, unit #2 to the signal line #2 and unit #3 to the signal line #1. Signal line #3 remains free (in reserve). On the (i+2)th full cycle of the system operation, a change of working signals is performed (Figure 4c). Unit #1 is connected to signal line #1, unit #2 to signal line #3 and unit #3 to signal line #4. Signal line #2 remains free (in reserve).

The operations of control and self-control of working capacity are the most important of them in light of the reliability requirements. At the same time, control packets of information are exchanged (with processing) between the PUs. A variant with sending, inverting the control word, returning this inversion to the supervisor, re-inverting and comparing with the original one is implemented in practice. If an error is detected (for example, a mismatch in the value of the control word), the PU supervisor turns off the functional PU and begins to perform its functions. The subsequent PU takes on the functions of a supervisor already with a new functional PU and so on.

**Figure 5:**
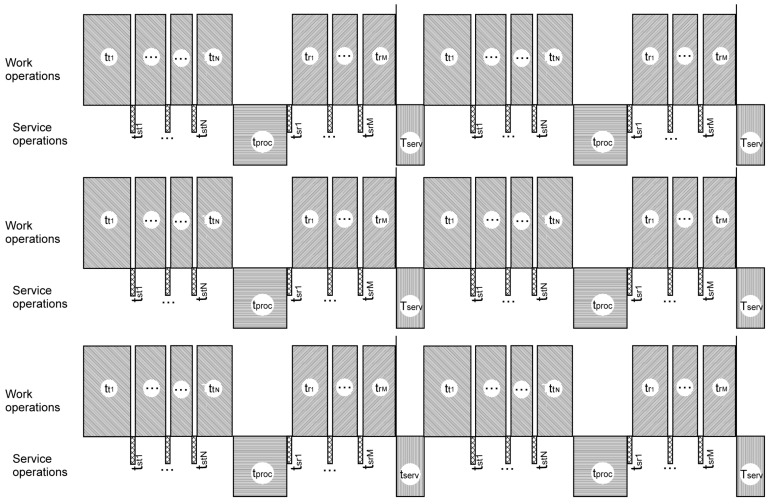
Timeline of work of the existing information exchange system embodied in real aircrafts. The blocks over the axis reflect working (useful) periods of work of other avionics. The blocks below the axis designate inner service periods of the information exchange system, with other avionics idle. Scales of blocks are chosen arbitrarily, for illustration purposes only.

**Figure 6:**
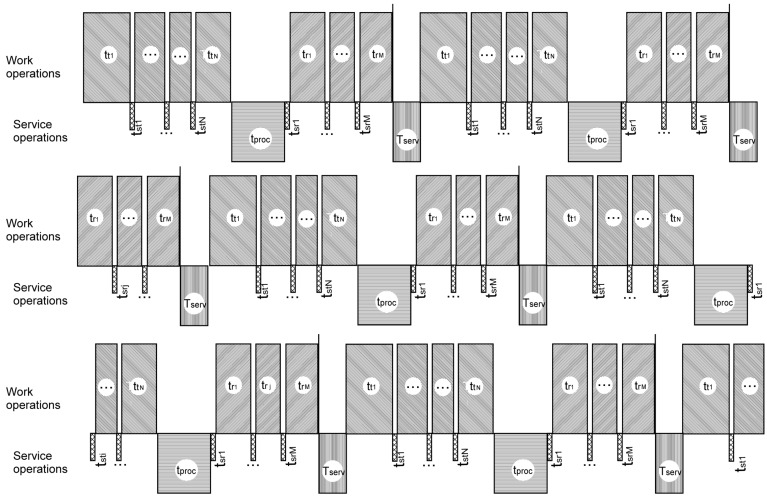
Timeline of work of the proposed information exchange system. Idle periods on each signal line are covered by work periods on other signal lines. The speed of transmitting/receiving signals by each information component is multiplied by the number of signal lines in comparison with the existing architecture.

The comparison of timelines of the existing system described in Figure 3 and the proposed architecture of the IXS units shown in Figure 4 are presented in Figure 5 and Figure 6, respectively.

**Figure 7:**
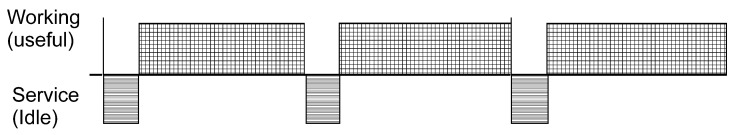
The summary time balance showing the drastic decrease of idle periods in the proposed architecture of the information exchange system.

Hatched blocks over the axis line correspond to “working” or “useful” periods of the on-board information system’s timeline, meaning that at least one of the components may transmit or receive information. The blocks below the axis line show “idle” or "useless” periods, when no component may transmit or receive signals. It can be clearly seen from Figure 6 that the idle periods on one line are covered by working periods on other lines in the proposed solution. The time of useless stay of the whole system is decreased to a possible minimum and even to zero in fortunate cases. Figure 7 presents the summary time balance showing the advantages of the proposed information exchange system.

## 4. Discussion

Since the main purpose of the study is to ensure the uninterrupted process of information exchange on board an aircraft, one of the key problems in this respect is to control all the components of the avionics for the presence of failures and to eliminate them if they occur or to reduce their impact to a possible minimum. In view of the extreme heterogeneity of the avionics components and their functions, the solution to the problem should concern affecting both hardware and software. According to the classification of avionics components, the possible sources of failures can be divided into the following ones:

(a) Power supply;

(b) Signal transmitters;

(c) Signal receivers;

(d) Signal lines;

(e) Hardware of IXS units itself;

(f) Software of IXS units itself;

(g) Interference induced in signal lines and avionics components either by external electrical-magnetic fields or by interference of avionics components;

(h) Erroneous actions of the aircraft crew or the ground operator in a case of an unmanned aerial vehicle.

The only possible way to improve flight safety is to duplicate the components themselves or their functions. In relation to the subject of the present study, this is the improvement of the IXS operation algorithm and, possibly, those addressees with which IXS interacts. It was stated above that the new algorithm of IXS operation allows us to increase the actual (total) speed of information exchange by a multiple of the number of units of IXS. In turn, this creates a temptation to spend some of the saved cycle time on additional component health checks. So, let us consider the features of the functioning of information exchange addressees and the capabilities of IXS for their control and elimination of failures. First of all, regardless of the functional purpose, all addressees alternately transmit/receive electrical signals via the same signal line(s). Moreover, all the transmitters (signal sources) are fundamentally divided into two categories: active and passive participants in information exchange. This implies that the active participant is able to receive a certain amount of information from the IXS, process it and send the already processed, modified amount of information back to the IXS. In the conditions of a real aircraft, these may include, for example, an electronic engine control system or other systems equipped with their own computing devices. Passive addressees include all the sensors and measuring devices that convert various physical quantities into an electrical signal. In this case, the only control element of such a component is a locking transistor (valve) that unlocks/locks the output of this addressee to a common signal line by the command IXS. Because of this, the set of tools for diagnosing a passive addressee is minimal. In practice, as a rule, this comes down to checking the presence/absence of a signal. However, in a number of cases, especially in the case of complex failures, the possibility of issuing false signals is not excluded. For example, parasitic currents induced in the electrical circuit can be taken as a useful signal of a sensor that actually failed. Moreover, the supply voltage of the sensor or other on-board systems can be output instead of the useful signal of the sensor in the case of physical damage to the signal line. For example, this can be caused by the sensor heating systems which prevent aircraft icing.

Two additional verification algorithms can be proposed under such conditions: a test for evidence “freezing” and a test for “absurdity”. The fact is that in real flight conditions, no measurable physical quantity can be constant. If it is conditionally considered constant, then in reality it fluctuates around a certain average value. Measuring sensors based on the conversion of any physical quantity into an electrical signal, as a rule, have a high degree of accuracy, which is enough to record possible fluctuations of the measured quantity. Thus, the proposed algorithm for checking for data “freezing” consists in storing the sensor readings in the previous cycle (cycles) of exchange in the IXS unit memory, calculating the difference between the current and previous sensor readings and comparing the result with a certain preset threshold value. If the result is zero, the given addressee is marked with a control “flag” (bit) as the „suspicious” one and its readings are monitored during the control number of cycles. If during this time the difference never exceeds the set threshold value, this addressee is considered as the failed one. In such a case, IXS can disconnect it from the signal line, either logically or physically. The logical method involves sending a constant blocking logic signal to the output transistor (gate) of the failed addressee. If there is a threat that the failure of the addressee can provoke the failure of the entire signal line, for example, the generation of a constant false signal with the simultaneous failure of the shut-off valve, then an additional possibility of physically disconnecting the failed addressee should be provided. This can be done, for example, in the form of pre-installation of an electromagnetic relay, with an appropriate power supply and a separate communication line to the IXS. Then the IXS failover algorithm should additionally include checking for the absence of signaling after sending the command to disconnect the addressee. If the test result is negative, then a command is sent to activate the electromagnetic relay.

A similar algorithm is proposed to test for “absurdity”. Its essence lies in the fact that any measured physical quantity can take values within a certain realistic range under real flight conditions. A value beyond it is considered “absurd”, that is, physically impossible, and indicates an addressee’s failure. It must be set taking into account all regular and abnormal situations in flight and even multiplied by the “ignorance coefficient”. This is necessary to avoid false positive failure alarms. For example, an “absurd” reading of a temperature sensor may indicate not its failure, but an actual fire on board. Similarly, an “absurd” reading of an acceleration or vibration sensor may indicate not a sensor failure, but the destruction of the aircraft structure, or other abnormal situations. In addition to the absolute value of the measured parameter, the behavior of this parameter, such as the first and second derivatives of the change in its value, or, for example, frequency, may also be informative in the sense of signaling an addressee failure in some cases. An additional algorithm which may be conditionally called cross-validation can be proposed, taking advantage of the newly proposed IXS architecture. It can be performed with respect to signal lines, as well as those components of on-board radio-electronic equipment that are critical from the point of view of flight safety, for example, control systems or power plants. The proposed cross-validation algorithm is as follows. At the moment of time $t_{k}$ the unit IXS${}_{i}$ receives the value of parameter $P_{j}$ from the *j*th checked device via the signal line $L_{m}$, dedicated to the IXS${}_{i}$ at the time moment $t_{k}$. After that, it sends the received value via the inner circuit of connection between IXS units to the unit IXS${}_{i+1}$ and unit IXS${}_{i+2}$. Further, at the moment of time $t_{k+1}$ the unit IXS${}_{i+1}$ receives the value of parameter $P_{j+1}$ from the *j*th checked device via the signal line $L_{m+1}$, dedicated to the IXS${}_{i+1}$ unit at the moment of time $t_{k+1}$. Upon this, unit IXS${}_{i+1}$ calculates the value $\Delta p_{j}=P_{j+1}-P_{j}$. If ${\left(\Delta p_{j}\right)}^{2}<A_{\mathrm{thd}}$, where $A_{\mathrm{thd}}$ is the some threshold value dedicated for the parameter $P_{j}$, the *j*th device is considered as a normal working one. In the other case, the *j*th device is considered as "failed" or "suspicious" according to the algorithm of failure processing chosen specially for the mentioned *j*th device. If the duration of the IXS cycle is variable, that is $T_{j}\neq T_{j+1}$, then ${\left(\Delta p_{j}\right)}^{2}/\left(T_{j+1}-T_{j}\right)$ should be checked as the rate of change of this parameter. Naturally, in each particular case, one should be guided by the specific features of each particular addressee of information exchange and adherence to good flight practice.

In addition to the above, an additional verification algorithm can be applied for active addressees, based on the presence of a computing device present there and, accordingly, able to process data. The algorithm is that IXS sends a control set of signals to the *j*th addressee, for example, a control word. The *j*th addressee must subject the received control word to a dedicated processing procedure—for example, shifting all bits in registers to the right or left, or, for example, inverting the received control word and sending the result back. IXS produces reversed arithmetic operations on the received response control word with respect to those operations that were performed by the addressee’s computing device, then compares the result with the original control word. The equality of the initial control word and the obtained result means that the *j*th addressee is serviceable.

The situation is more complicated with the verification of addressee-signal receivers. Obviously, each such addressee must have the technical ability to communicate with the IXS unit. If the addressee is an information component, it must be equipped with an additional processing or logical unit capable of converting and transmitting the received control word or other signal. If the receiver is some kind of mechanical device that operates on commands received from the IXS (for example, a fuel system throttle, a rudder drive, etc.), then in order to check its performance, it must be equipped, firstly, with a sensor capable of measuring the characteristic physical quantity, which is decisive for its performance and then converting it into an electrical analog or digital signal. Such a characteristic physical quantity can be linear or angular displacement, force, liquid or gas flow rate, orbital frequency, etc. Secondly, it must be equipped with a transmitter capable of transmitting this signal to the IXS. From the point of view of the functioning of IXS, such a device should be considered as a receiver transmitter which receives a command from the IXS in the receive subcycle. It performs the transmission to the IXS of the measured result of the previously sent command in the transmission subcycle. Furthermore, compensation for lost functions is required in addition to exclusion of the failed addressee from the work. If the addressee has been reserved, its functions are transferred to an alternative that does not have a failure. If it was not duplicated, or all alternatives also fell into a failure state and the signals received from this addressee are absolutely necessary for piloting the aircraft, the IXS will substitute the missing value of the received values, either by the last correct value received from the failed addressee, or by some pre-assigned default value.

Mutual control of the IXS units themselves should be performed according to the algorithm for active transmitters. In [19], it is proposed to monitor the performance and to exclude a failed active component from operation, performing the “voting” method. This assumes the presence of two or more serviceable units against one failed. Here we should pay attention to the case when only two units remain serviceable. The implementation of the above algorithm is possible only if the block being checked is hierarchically subordinate to the second, supervisor block, which is in hot standby and is a priori considered to be serviceable. However, all the units are equal and simultaneously participate in the information exchange process according to the IXS units’ architecture discussed in this paper. So the following algorithm is proposed for such a case. A blocking transistor (valve) is installed on the output to signal line of each of the IXS units, which is connected to the rest of the IXS units via a separate communication line. Cyclically, e.g., at the end of each complete operating cycle of the entire IXS system, or at another moment, each IXS unit sends lock signals to the inputs of the locking transistors of all other IXS unit outputs. At the same time, it sends a control signal via the internal communication circuit between IXS units. A mutual synchronization signal can be used instead of a separate control signal, for example. Having received a control signal, a serviceable IXS unit sends the unlocking signal to the input of its locking transistor, which unlocks it, opening the output of this IXS unit to the signal line. In contrast, if this IXS unit is faulty, then it is unable either to receive a control signal or to open the locking transistor. Hereinafter, it remains isolated from the signal lines and does not interfere with the information exchange. The proposed version of the IXS architecture is completely based on the reliable existing hardware, does not change any already existing safety measures and system reserving and meets all the requirements of existing standards and civil aviation regulations. The shortcoming of the proposed solution consists in “asymmetry” working mode, i.e., time intervals between cycles are not equal. In the future improvement of the IXS architecture, the operational cycle of the IXS should be shortened in order to raise the information exchange speed. 

## 5. Conclusions

We have proposed the modification of architecture and algorithms of the aircraft on-board information exchange system. The IXS operation algorithm should include all the possible failure modes, up to the failure of (N−1) IXS units and *N* data buses. In addition, the computational performance of the IXS unit must be selected to provide the minimum allowable data exchange rate in the event of failure of (N−1) IXS units. The proposed solution allows us to increase the speed of information exchange supported by the IXS in accordance with the number of IXS units, at the same time maintaining the reliability indicators inherent in the previously known solutions and on the same element base. It is also possible to solve the inverse problem, i.e., maintaining the original performance of the IXS with a multiple decrease in the required computing performance of blocks accompanied with a corresponding reduction of mass, volume and power consumption. Naturally, the proposed algorithm requires in-depth study, research by simulation methods as well as tests in bench conditions and on board an aircraft.

## Data Availability

Not applicable.
